# Insulin degludec and insulin glargine 300 U/mL: Which of these two insulins causes less hypoglycemia?

**DOI:** 10.1111/jdi.13065

**Published:** 2019-05-28

**Authors:** Silvio Buscemi, Cristiana Randazzo, Carola Buscemi

**Affiliations:** ^1^ Unit of Endocrinology, Metabolic and Nutrition Diseases University Hospital Policlinico “P. Giaccone” – University of Palermo Palermo Italy; ^2^ Unit of Geriatrics Garibaldi‐Nesima Hospital Postgraduate School of Geriatrics University of Catania Catania Italy

## Abstract

Insulin degludec versus insulin glargine 300 U/mL.

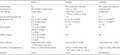

The interesting article by Yamabe *et al*.^1^ showed, using continuous glucose monitoring, that insulin degludec (I‐Deg) was associated with a high percentage of time with nocturnal hypoglycemia than with insulin glargine 300 U/mL (I‐G300; *P* = 0.02). However, we observe that some possible confounding factors might have influenced the results, such as differences in concomitant medications, use of the same titration protocol for both kinds of insulin or differences in glucose levels. This is also a recurrent problem in clinical trials, which sometime produce conflicting results. In fact, the study of Yamabe *et al*. is partly in agreement with some recently published clinical trials that gave different conclusions. In the last months of 2018, three studies^2^, ^3^, ^4^ compared I‐Deg with I‐G300 using different approaches, but their conclusions were quite different, especially regarding the possibility of inducing hypoglycemia.

In Clinical Outcome Assessment of the Effectiveness of Insulin Degludec in Real‐life Medical Practice (CONFIRM)^3^, a real‐world study, the use of I‐Deg versus I‐G300 was associated with a lower risk of hypoglycemia. Surprisingly, the other two recent comparative studies, Differentiate Gla‐300 clinical and Economic in Real‐World Via EMR (DELIVER)^3^ and BRIGHT^4^, led to opposite conclusions (Table 1). CONFIRM and DELIVER had very different study designs (one above all, was the insulin naive *vs* switch procedure); in both studies the basal glycated hemoglobin was high, and most participants did not achieve the optimal glucose control, all conditions that make it difficult to made an adequate assessment of the risk of hypoglycemia. In contrast to CONFIRM, DELIVER also included hypoglycemic events observed in the emergency department. The reliability in reporting hypoglycemic events is another important confounding factor, especially in real‐world evidence studies. Regarding the BRIGHT study, it was found that the risk of hypoglycemia was slightly, but significantly, higher with the use of I‐Deg than with I‐G300, especially during the phase of titration (hypoglycemia ≤70 mg/dL: +2.39 events per patient‐year, *P* = 0.02; hypoglycemia ≤54 mg/dL: +0.37 events per patient‐year, *P* = 0.04). However, according to the data presented in table 1 of reference 4 of the BRIGHT study (significance level was not reported), the two groups were not comparable. In fact, applying the Student's *t*‐test for unpaired data, we observed that patients allocated to the I‐Deg group had significantly (*P* < 0.01) lower glycated hemoglobin values than those of the I‐G300 group (8.57 vs 8.71%), but also significantly (*P* < 0.01) lower fasting plasma glucose concentrations (182 vs 191 mg/dL) and self‐monitoring plasma glucose (172 vs 178 mg/dL; *P* < 0.05). Also, the authors reported that by using fewer units of I‐Deg (0.43 units/kg) than I‐G300 (0.54 units/kg), fasting plasma glucose concentrations were more reduced in the I‐Deg group, with a significant difference of 7.68 mg/dL, versus the I‐G300 group. These results would suggest that I‐Deg probably has a pronounced hypoglycemic power. Another important confounding factor is the different use of concomitant medications between the studies, in particular that of secretagogues, such as sulfonylureas. Given the different direct and indirect (mainly due to hypoglycemia) costs of I‐Deg and I‐G300, it is important to clarify these controversial aspects. In conclusion, we believe that either head‐to‐head randomized controlled trials or real‐world studies need to be designed including well‐matched groups, paying special attention to potential confounding factors. Otherwise, the risk is to prove everything and nothing.

**Table 1:** Characteristics of the three studies that compared insulin degludec versus insulin glargine 300 U/mL

	BRIGHT	DELIVER	CONFIRM
Study design	RCT	RW, propensity matched	RW, propensity matched
Participants (*n*)	463 (I‐G300) vs 466 (I‐Deg)	1,592 vs 1,592	2,028 vs 2,028
T2D patients	Insulin naive	Switch from I‐G100 or I‐Det to I‐G300 or I‐Deg	Insulin naive
Use of sulphonylureas (%)	~65	~25	~25
Basal HbA1c (%)	8.71 ± 0.83 (I‐G300)	9.1 ± 1.8 (I‐G300)*	9.6 ± 2.2 (I‐G300)**
	8.57 ± 0.80 (I‐Deg)	9.1 ± 1.9 (I‐Deg)*	9.5 ± 2.1 (I‐Deg)**
	*P* < 0.001	*P* = NS	*P* = NS
FPG (mg/dL)	191 ± 49 (I‐G300)	NA	NA
	182 ± 51 (I‐Deg)		
	*P* < 0.001		
SMPG (mg/dL)	178 ± 40 (I‐G300)	NA	NA
	172 ± 38 (I‐Deg)		
	*P* < 0.001		
Insulin efficacy	I‐Deg better than I‐G300 (~20% fewer units of I)	I‐Deg similar to I‐G300	I‐Deg better than I‐G300
Incidence of hypoglycemia	I‐Deg > I‐G300 (titration phase)	I‐Deg > I‐G300 (event rate PPY: 0.08 vs 0.05; *P* = 0.016)	I‐Deg < I‐G300 (–30% risk)
Sponsor	Sanofi	Sanofi	NovoNordisk

Data presented as the mean ± standard deviation. Statistical comparison between studies and for data of the BRIGHT study was carried out using the Student's unpaired *t*‐test: *P < 0.001 versus BRIGHT; **P < 0.001 versus Differentiate Gla‐300 clinical and Economic in Real‐World Via EMR (DELIVER) and BRIGHT. CONFIRM, Clinical Outcome Assessment of the Effectiveness of Insulin Degludec in Real‐life Medical Practice; FPG, fasting plasma glucose; G, glargine; HbA1c, glycated hemoglobin; I‐Deg, insulin degludec; I‐Det, Det, insulin detemir; I‐G300, glargine 300 U/mL; NA, not available; NS, not significant; PPY, per person‐year; RCT, randomized controlled trial; RW, real‐world; SMPG, self‐monitoring plasma glucose; T2D, type 2 diabetes.

## Disclosure

The authors declare no conflict of interest.
